# Pharmacological Inhibition of Caspase-2 Protects Axotomised Retinal Ganglion Cells from Apoptosis in Adult Rats

**DOI:** 10.1371/journal.pone.0053473

**Published:** 2012-12-28

**Authors:** Vasanthy Vigneswara, Martin Berry, Ann Logan, Zubair Ahmed

**Affiliations:** Neurotrauma and Neurodegeneration Section, School of Clinical and Experimental Medicine, College of Medical and Dental Sciences, University of Birmingham, Birmingham, United Kingdom; Schepens Eye Research Institute, Harvard Medical School, United States of America

## Abstract

Severing the axons of retinal ganglion cells (RGC) by crushing the optic nerve (ONC) causes the majority of RGC to degenerate and die, primarily by apoptosis. We showed recently that after ONC in adult rats, caspase-2 activation occurred specifically in RGC while no localisation of caspase-3 was observed in ganglion cells but in cells of the inner nuclear layer. We further showed that inhibition of caspase-2 using a single injection of stably modified siRNA to caspase-2 protected almost all RGC from death at 7 days, offering significant protection for up to 1 month after ONC. In the present study, we confirmed that cleaved caspase-2 was localised and activated in RGC (and occasional neurons in the inner nuclear layer), while TUNEL^+^ RGC were also observed after ONC. We then investigated if suppression of caspase-2 using serial intravitreal injections of the pharmacological inhibitor z-VDVAD-fmk (z-VDVAD) protected RGC from death for 15 days after ONC. Treatment of eyes with z-VDVAD suppressed cleaved caspase-2 activation by >85% at 3–4 days after ONC. Increasing concentrations of z-VDVAD protected greater numbers of RGC from death at 15 days after ONC, up to a maximum of 60% using 4000 ng/ml of z-VDVAD, compared to PBS treated controls. The 15-day treatment with 4000 ng/ml of z-VDVAD after ONC suppressed levels of cleaved caspase-2 but no significant changes in levels of cleaved caspase-3, -6, -7 or -8 were detected. Although suppression of caspase-2 protected 60% of RGC from death, RGC axon regeneration was not promoted. These results suggest that caspase-2 specifically mediates death of RGC after ONC and that suppression of caspase-2 may be a useful therapeutic strategy to enhance RGC survival not only after axotomy but also in diseases where RGC death occurs such as glaucoma and optic neuritis.

## Introduction

Injury to the optic nerve (ON) triggers progressive death of retinal ganglion cells (RGC), the severity of which is dependent upon the type of lesion and its distance from the eye [1], [2], [3]. For example, intraorbital ON transection and ON crush (ONC) both trigger 70–75% RGC loss within 7 days after injury [4], [5], [6], [7] and by 28 days, 80–90% RGC are lost, primarily by apoptosis [8], [9], [10]. RGC apoptosis, however, is recognised as a limiting factor to the regenerative potential of RGC axons. Therefore, treatments to block RGC apoptosis have been studied extensively. For example, inhibition of apoptosis by neurotrophic factor administration [11], overexpression of Bcl-2 [12], [13] and inhibition of caspase-1 and -3 [14], [15] and caspase-6 and -8 [16] using pharmacological inhibitors all reduced the number of dying RGC after ON transection and ONC. To date, only caspase-6 and -8 inhibitors have yielded limited RGC axon regeneration after ON axotomy [16].

Apoptosis is orchestrated by caspases, cysteine-rich proteases capable of targeting proteins that play critical roles in DNA replication [17], [18], DNA repair [19], cell survival signalling [20] and the regulation of proteins that control cytoskeletal re-organisation and cellular disassembly [21], [22]. There are two groups of caspases: initiator (caspase-2, -8, -9, and -10) and effector caspases (caspase-3, -6 and -7) the former are activated by either death receptor activation, or the release of cytochrome-c from mitochondria, which activate effector caspases through proteolytic processing of pro-caspases, culminating in cleavage of structural proteins and eventual death [23], [24], [25], [26].

One of the most highly conserved caspases is caspase-2, which acts as both an initiator and an executioner depending on the apoptotic stimuli [27], [28], [29], [30]. Caspase-2 deficient neurons are resistant to apoptosis by β-amyloid [31], [32] while activation of caspase-2 mediates apoptosis of hippocampal neurons after transient global ischemia [33]. Caspase-2 is also expressed in the RGC of ischaemic retinae [34] and the neuroprotective effect of brain-derived neurotrophic factor (BDNF) is associated with reduced caspase-2 [35]. We have shown unequivocally that, 7 days after ONC, caspase-2 is specifically activated in RGC and that inhibition of caspase-2 by stably-modified siRNA protects 98% of RGC from death at 7 days after ONC and significant RGC protection lasted for at least 30 days [5], [6], [7]. Here, we report that a serially injected cell permeable pharmacological inhibitor of caspase-2 protects 60% of RGC from apoptotic death 15 days after ONC but does not promote RGC axon regeneration. Our results suggest that caspase-2 is an important executioner molecule in RGC apoptosis.

## Materials and Methods

### Ethics statement

This study was carried out in strict accordance to the Animals Scientific Procedures Act, 1986 and all procedures were licensed and approved by the UK Home Office. The protocols and experiments were also approved by the University of Birmingham Ethical Review Sub-Committee. Animals were kept in environmentally controlled animal facilities at the University of Birmingham. All surgery was performed under inhalation anaesthesia using 5% Isofluorane (IsoFlo, Abbott Animal Health, North Chicago, IL, USA) induction and 2% for maintenance. Every effort was made to minimise animal suffering.

### ON crush (ONC)

The ON of adult female 200–250 g Spraque-Dawley rats (Charles River, Margate, UK) was exposed through a supraorbital approach and crushed bilaterally within the orbit, 2 mm from the eye, using forceps as described previously [5], [7], [36], [37], [38], [39].

### Intravitreal injections

The cell membrane permeable caspase-2 inhibitor, z-V-D-(OMe)-V-A-D(OMe)-fluromethylkeone (z-VDVAD) (R&D Systems, Abingdon, Oxford, UK), was dissolved in sterile DMSO (Sigma, Poole, UK) and further diluted in sterile PBS before intravitreal injection in a final volume of 5 µl. Control animals (n = 6 rats/treatment (12 eyes/treatment)) were intravitreally injected with PBS (containing the same diluted amount of DMSO as z-VDVAD) (vehicle) at the same time-points as z-VDVAD, whilst treated rats (n = 6 rats/treatment (12 eyes/treatment)) received either 400 ng/ml, 1000 ng/ml, 2000 ng/ml, 4000 ng/ml or 5000 ng/ml of z-VDVAD immediately after ONC (day 0), and intravitreal injections repeated at 4, 8 and 12 d after ONC. The rationale for doses of 400 ng/ml and 4000 ng/ml were based on previously published data using caspase-3 inhibitors [40]. Animals survived for 15 d, after which the retinae were dissected out for retinal wholemounts and protein extraction for western blotting, while whole eyes were removed for immunohistochemistry. None of the animals developed cataracts, confirming that the lens had not been injured either during surgery or after subsequent intravitreal injections.

### Determining the optimal dosing regime

To determine the frequency of intravitreal z-VDVAD injections, we used the same dose of a pharmacological inhibitor to caspase-3 and the same 3–4 d injection schedule after ONC, reported by Kermer et al. (1998) [40] that caused RGC survival. We therefore chose to intravitreally inject 4000 ng/ml immediately after ONC and killed animals at 2, 3 and 4 days by overdose of CO_2_ (n = 3 rats/treatment (6 retinae/treatment)). Eyes were enucleated, retinae harvested and snap frozen until required for extraction of total proteins and western blotting as described later.

### Retinal wholemounts

At 13 d after ONC, 2 µl of 4% FluoroGold (FG, Cambridge Bioscience, Cambridge, UK), were prepared from a solid stock and injected into the proximal ON segment mid-way between the lamina cribrosa and the site of ONC. Animals were killed and eyes were enucleated 48 h later and retinae were immersion-fixed in 4% formaldehyde (TAAB Laboratories, Aldermaston, UK) for 30 min, and flattened onto a Superfrost Plus microscope slides (VWR International, Lutterworth, UK), after dividing the retinae with 4 equidistant radial cuts to give 4 equally sized quadrants attached together at the optic disc. Retinal wholemounts were dried onto glass slides and mounted in Vectamount (Vector Laboratories, Peterborough, UK). Samples were randomised and blinded by a second investigator and photographs were captured using a Zeiss fluorescent microscope equipped with a digital camera in Axiovision 4 (all from Zeiss, Hertfordshire, UK). The number of FG-labelled RGC was counted using automated particle counting software in ImagePro Version 6.0 (Media Cybernetics, Bethesda, USA) from photographs of 12 rectangular areas (0.36×0.24 mm), 3 from each quadrant, placed at radial distances from the centre of the optic disc of the inner (1/6 eccentricity), midperiphery (1/2 eccentricity) or outer retina (5/6 eccentricity). RGC densities were summed together and averaged over the entire retina and expressed as mean RGC densities/mm^2^ for each treatment (n = 6 rats/treatment (12 retinae/treatment)).

### Tissue preparation and sectioning

After intracardiac perfusion with 4% formaldehyde, eyes and ON were removed, immersion-fixed in 4% formaldehyde (TAAB) for 2 h, washed for 10 min in 10 mM phosphate buffered saline (PBS), and immersed in 10% and 20% sucrose (Sigma) each for 2 h and finally immersed in 30% sucrose overnight. Eyes and ON were embedded in OCT mounting medium (Raymond A Lamb Ltd) and 15 µm thick parasaggital sections of eyes and longitudinal sections of ON were cut on a cryostat (Bright Instruments, Huntingdon, UK), adhered onto glass slides and stored at −20°C until required.

### Immunohistochemistry

Double immunohistochemistry for cleaved caspase-2 (C-CASP2) and βIII-tubulin was performed on sections of retina as described by us previously [7]. Briefly, sections were washed in PBS, non-specific binding blocked with PBS containing 3% BSA and 0.05% Tween 20 for 20 min before incubation with rabbit anti-C-CASP2 (Abcam, Cambridge, UK; 1∶200 dilution) and monoclonal anti-βIII-tubulin (Sigma, Poole, UK; 1∶200 dilution) primary antibodies overnight at 4°C. Sections were then washed in ×3 changes of PBS, incubated with appropriate Alexa Fluor 488 and Texas Red-labelled secondary antibodies for 1 h at room temperature, washed, mounted using Vectashield mounting medium with DAPI (Vector Laboratories) and examined under an Axioplan-2 epi-fluorescent microscope (Zeiss).

GAP-43 immunohistochemistry was performed on ON sections (n = 9 rats/treatment; (i.e. 18 ON)) to detect RGC axon regeneration at 15 d after ONC using a sheep polyclonal anti-GAP-43 antibody (donated by Professor Larry Benowitz, Harvard Medical School, Boston, USA) as described previously [39].

### Protein extraction and Western blotting

At 15 d after ONC, a total of 3 rats/treatment group were killed and retinae (n = 3 rats/treatment (6 retinae/treatment)) were pooled prior to protein extraction in cell lysis buffer and processed for Western blotting as previously described [7]. Experiments were then repeated×2 using a further 3 rats/treatment/experiment and retinae (n = 6 retinae/treatment/experiment) were again pooled and proteins extracted prior to western blot analysis. Western blots were probed overnight at 4°C with antibodies against: Goat anti-human cleaved caspase-2 (directed against the p12 fragment) (C-CASP2) and rabbit anti-human cleaved caspase-8 (p20 fragment) (C-CASP8) (both from Santa Cruz Biotechnology, CA, USA); Rabbit anti-cleaved caspase-3 (C-CASP3), rabbit anti-cleaved caspase-6 (C-CASP6) and rabbit anti-cleaved caspase-7 (C-CASP7) (all from Cell Signalling Technology, Danvers, MA, USA). Relevant bands were detected with an appropriate HRP-labelled secondary antibody (GE Healthcare, Buckinghamshire, UK) and detected using an enhanced chemiluminescence system (ECL) (GE Healthcare).

Western blots were probed with a rabbit monoclonal antibody to GAPDH (Cell Signalling Technology, Danvers, MA, USA) at 1∶1000 dilution and used as a loading control. Blots were stripped and re-probed as required.

### Biotin-VAD-fmk trapping assay of active caspase

The caspase trapping assay which employs the bVAD (biotin-Val-Ala-DL-Asp-fluoromethylketone (biotin-VAD-fmk)) probe is the best way to determine whether caspases are active after a death stimuli. bVAD is an irreversible pan-caspase inhibitor and binds irreversibly to all activated caspases. Once bVAD binds irreversibly it inhibits that specific caspase and blocks downstream events, and as the bVAD is biotinylated, it can be isolated on streptavidin agarose along with any active caspases that are bound to it. We therefore used the biotin-VAD-fmk assay was used to detect the presence of active caspase-2 as described previously [28], [41], [42] but with modifications for our *in vivo* experiments. Briefly, 200 nmol of b-VAD-fmk (MP Biomedical, UK) was diluted in 5 µl of sterile saline and intravitreally injected 24 hr prior to ONC (to capture any caspases being activated by ONC) while control animals received 5 µl of sterile saline prior to ONC. bVAD treated animals were killed at 1, 4 and 7 d after ONC whilst control animals were killed at 4 d (since our previous experiment demonstrated optimal C-CASP2 immunohistochemistry at this time-point). Eyes were enucleated and retinae were immediately homogenised in ice-cold CHAPS buffer (150 mM KCl, 50 mM HEPES and 0.1% CHAPS (pH 7.4)) supplemented with protease inhibitor cocktail (Sigma). Samples were incubated on ice for 10 min prior to centrifugation at 13000× g for 10 mins at 4°C and the supernatants were collected and boiled for 5 min. Streptavidin-agarose beads were then added to the boiled supernatant and incubated overnight at 4°C with constant agitation. Beads were spun at 7500× g for 5 min at 4°C, washed ×5 in PBS, resuspended in 2× Laemmli buffer and boiled for 5 min. The beads were centrifuged at 15000× g for 10 min, supernatant collected and supplemented with 5% 2-mercaptoethanol and samples were resolved on 12% SDS-PAGE gels and analysed by western blotting as described above using an affinity purified goat polyclonal anti-C-CASP2 antibody (Santa Cruz Biotechnology). Blots were stripped and re-probed with antibodies against total CASP-2 (Santa Cruz Biotechnology) and GAPDH (Cell Signalling Technology).

### Densitometry

Western blots were quantified by densitometry as described by us previously [7], [38], [39]. Briefly, blots were scanned into Adobe Photoshop and TIFF files were analysed in ScionImage (version 4.0.2, Scion Corp, Maryland, USA) using the built-in gel plotting macros. The integrated density of each band in each lane was calculated from 3 separate blots.

### Statistical analysis

The significance of differences between sample means were calculated using GraphPad Prism (GraphPad Software Inc., Version 4.0, CA, San Diego, USA) by one-way analysis of variance (ANOVA) followed by *post-hoc* testing with Dunnett's method.

## Results

### ONC induced caspase-2 activation and TUNEL in RGC

**Figure 1:**
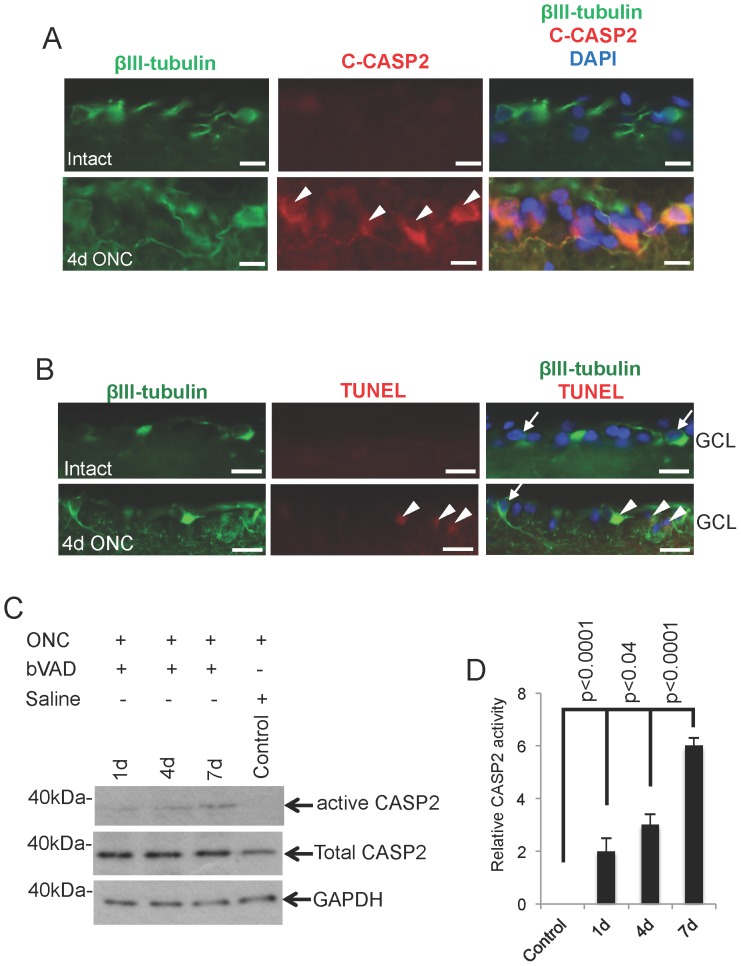
Caspase-2, TUNEL localisation in RGC and caspase trapping assay. (A) Immunohistochemistry to show the absence of C-CASP2 (red) reactivity in βIII-tubulin^+^ (green) RGC in intact controls, while 4 days after ONC the majority of βIII-tubulin^+^ RGC (green) were C-CASP2^+^ (red; arrowheads). (B) βIII-tubulin^+^ RGC in intact controls were also negative for TUNEL staining while some TUNEL^+^ (arrowheads) RGC were present at 4 d after ONC. Some RGC were also negative for TUNEL staining at 4 d after ONC (arrow). (C) Active CASP2 is induced by ONC and its levels increase over the 7 d time period and (D) densitometry to quantify the relative CASP2 activity compared to controls. GCL = ganglion cell layer. Scale bars in A and B = 50 µm.

We have previously shown that C-CASP2 is localised in RGC within 5 hr after ONC and is present in a significant number of RGC after 24 hr [7]. Here we confirmed by immunohistochemistry that C-CASP2 was absent in βIII-tubulin^+^ RGC in intact (uninjured) animals (Figure 1A), but was specifically localised in RGC and present in abundant numbers of RGC at 4 d after ONC (Fig. 1A). The localisation of C-CASP2 paralleled the presence of TUNEL^+^ nuclei in βIII-tubulin^+^ RGC in ONC animals (Figure 1B), indicating RGC apoptosis. The b-VAD caspase trapping assay captured increasing amounts of CASP2 activity and demonstrated 2-, 3- and 6-fold higher levels of CASP2 at 1 d, 4 d and 7 d, respectively, when compared to controls (Figure 1C and D). Other caspases (e.g. CASP3 or CASP7 (data not shown)) were not captured. These results demonstrate that CASP2 is localised and specifically activated in RGC after ONC.

### Determining the frequency of z-VDVAD administration

**Figure 2:**
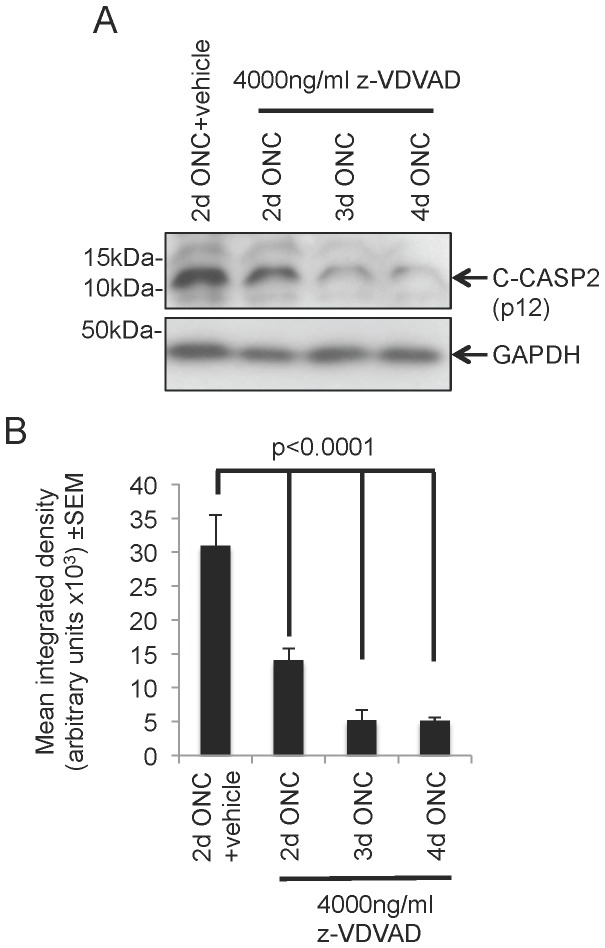
Determining the optimal z-VDVAD dosing regime. (A) Representative western blot to show that 4000 ng/ml z-VDVAD treatment caused optimal suppression of C-CASP2 (>80%) by 3 d after ONC. (B) Densitometry confirmed 50% reduction of C-CASP2 by 2 d after ONC+z-VDVAD treatment compared to ONC alone, which increased to 80% at 3 d after ONC+z-VDVAD treatment with no further reduction by 4 d. GAPDH was used as a loading control. n = 6 rats/treatment (12 eyes/treatment).

After ONC and immediate intravitreal injection of 4000 ng/ml of z-VDVAD, western blotting detected reduced levels of C-CASP2, of approximately 50% (P<0.0001, ANOVA), at 2 d after ONC compared to ONC+vehicle treated eyes (Figure 2A and B). The levels of C-CASP2 decreased significantly by >85% at 3 days (P<0.0001, ANOVA), remaining significantly low for 4 days (P<0.0001, ANOVA) but without further reductions from day 3 (Figure 2A and B). Based on these results, every 4^th^ day was selected for repeated intravitreal injections of z-VDVAD for the duration of the 15 d experimental time period to minimise the number of intravitreal injections required prior to harvesting of tissues.

### z-VDVAD promoted RGC survival after ONC

**Figure 3:**
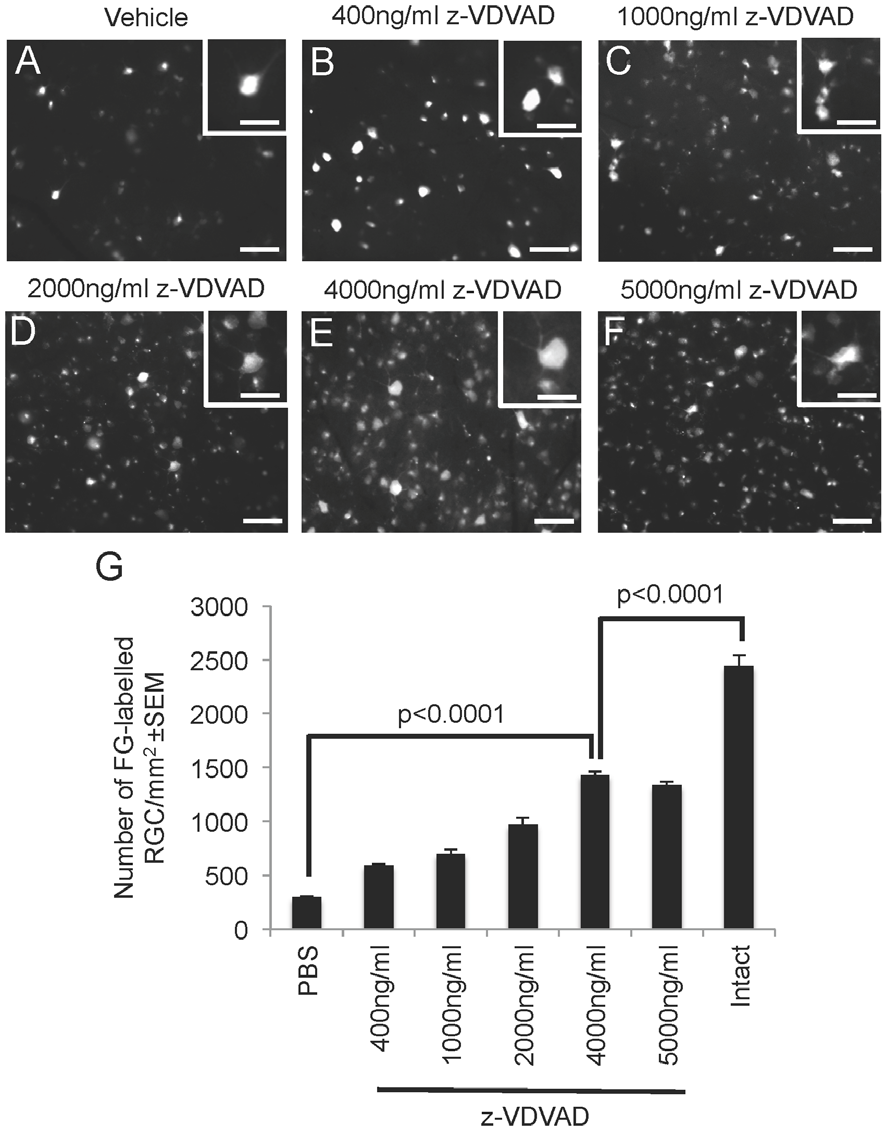
Treatment with z-VDVAD protects RGC from death at 15 d after ONC. FG^+^ RGC in flat mounted retina and high power inserts after (A) ONC+vehicle, (B) ONC+400 ng/ml z-VDVAD, (C) 1000 ng/ml and ONC+1000 ng/ml z-VDVAD, (D) ONC+2000 ng/ml z-VDVAD, (E) ONC+4000 ng/ml z-VDVAD, (F) ONC+5000 ng/ml z-VDVAD at 15 d after ONC. (G) Quantitation of surviving RGC density (±SEM) at 15 d after ONC and treatment with z-VDVAD. Scale bars in A–F = 50 µm, and in insets = 25 µm.

To determine whether inhibition of caspase-2 protected RGC from apoptosis for 15 d after ONC, we injected z-VDVAD intravitreally every 4^th^ day and compared results with control vehicle treated eyes. Very few FG^+^ RGC were observed in retinal wholemounts from ONC+vehicle injected eyes (Figure 3A and G) compared to those treated with increasing concentrations of z-VDVAD (400 ng/ml to 5000 ng/ml) (Figure 3B–F and G), where progressively more FG^+^ RGC were present up to a concentration of 4000 ng/ml (Figure 3E and G) (P<0.0001, ANOVA). There was no increase in FG^+^ RGC using the highest concentration of z-VDVAD (5000 ng/ml) (Figure 3F and G) compared to that observed at 4000 ng/ml RGC (Figure 3E and G). No changes in cell morphology, nor soma size were observed in retinal wholemounts after treatment with z-VDVAD, with all RGC morphology appearing healthy and as diverse soma sizes as that observed in controls without z-VDVAD treatment (Figure 3A–F: insets) The average density of FG^+^ RGC in ONC+vehicle treated eyes were 293±34 RGC/mm^2^ (Figure 3A). Intravitreal delivery of 400 ng/ml of z-VDVAD increased RGC survival to 590±56 RGC/mm^2^, while the optimum concentration of 4000 ng/ml z-VDVAD increased survival to 1430±30 RGC/mm^2^ (Figure 3G). Therefore, 4000 ng/ml of z-VDVAD protected 60% of RGC from death after 15 d compared to uninjured, intact controls (Figure 3G).

### z-VDVAD reduces caspase-2 activation in RGC of the ganglion cell layer

**Figure 4:**
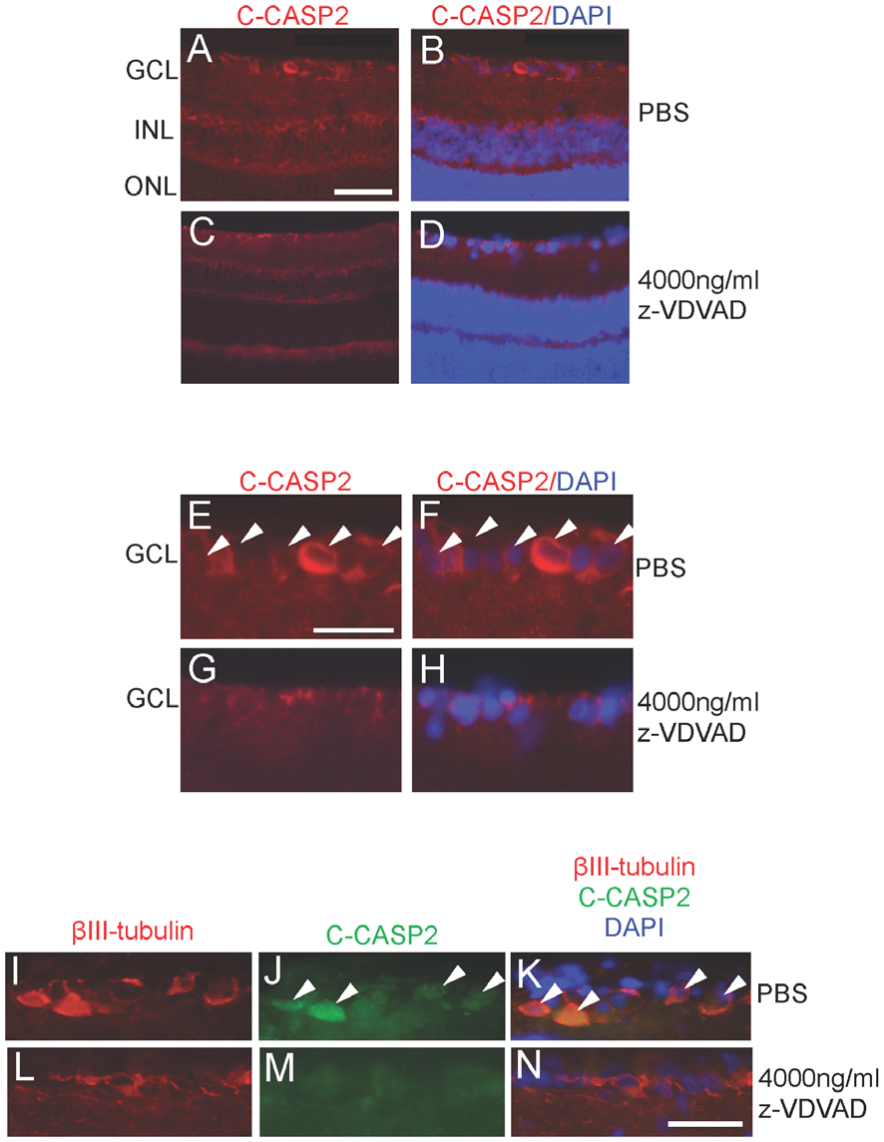
C-CASP2 immunoreactivity after treatment with z-VDVAD. (A, B) C-CASP2 is highly up regulated in ONC+vehicle treated in the GCL and INL cells. (C, D) C-CASP2 immunoreactivity (red) was barely detectable in the GCL after treatment with 4000 ng/ml z-VDVAD. (E–H) High power magnification of the GCL showed C-CASP2 immunoreactivity (red) in RGC after ONC+vehicle treatment (arrowheads) whilst treatment with z-VDVAD suppressed immunoreactivity in the GCL. Double immunohistochemistry showing that βIII-tubulin^+^ RGC (I) were also C-CASP2^+^ (J) (arrowheads) after ONC+vehicle treatment while treatment with ONC+4000 ng/ml z-VDVAD suppressed C-CASP2 levels in RGC (L–N). GCL = ganglion cell layer, INL = inner nuclear layer; ONL = outer nuclear layer. Scale bars in A–D, 100 µm, scale bars in E–N, 50 µm.

We investigated if intravitreal z-VDVAD injection maintained the suppression of cleaved caspase-2 (C-CASP2) in sections of retinae by immunohistochemistry at 15 d. After ONC+vehicle treatment (Figure 4A and B), C-CASP2 was localised in most ganglion cells (GCL) and cells of the inner nuclear layer (INL). However, treatment with 4000 ng/ml of z-VDVAD (Figure 4C and D) almost completely suppressed C-CASP2 immunohistochemistry. High power magnifications of the GCL confirmed C-CASP2 immunolocalisation in cells of the GCL after ONC+vehicle treatment (Figure 4E and F) while little or no immunolocalisation for C-CASP2 was observed in the cells of the GCL of eyes treated with 4000 ng/ml z-VDVAD (Figure 4G and H).

Co-localisation of C-CASP2 with βIII-tubulin^+^ confirmed that βIII-tubulin^+^ RGC showed specific C-CASP2 activation in RGC after ONC+vehicle (Figure 4I–K) but little or no C-CASP2 activation in βIII-tubulin^+^ RGC after treatment with 4000 ng/ml z-VDVAD (Figure 4L–N).

### Only C-CASP2 levels are suppressed by z-VDVAD

**Figure 5:**
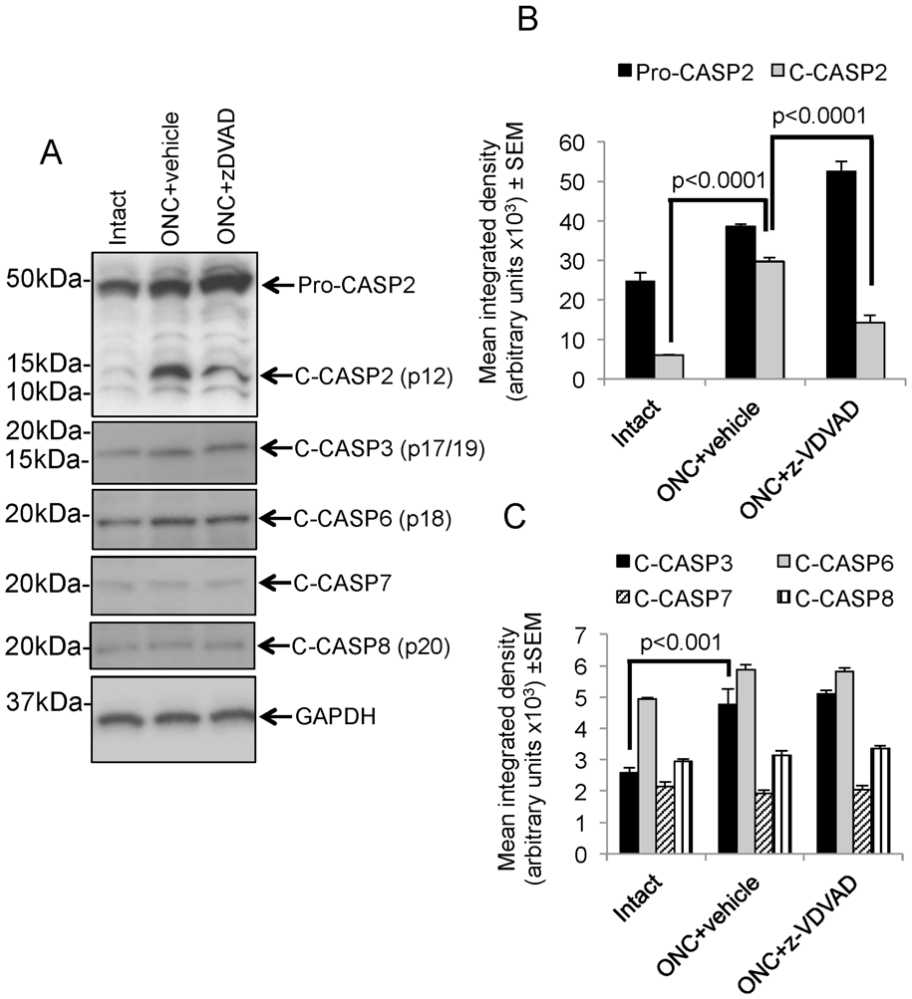
Western blot analysis after z-VDVAD treatment. (A) Pro-CASP2 levels rose after ONC and remained high even after treatment with 4000 ng/ml z-VDVAD. The level of the C-CASP2 p12 fragment also rose after ONC but was significantly decreased after treatment with z-VDVAD. (A) and (B) Levels of Pro-CASP2 and C-CASP2 (p12) reflected these changes and showed that the C-CASP2 fragment was 40% lower in retinae treated with 4000 ng/ml z-VDVAD than those treated with vehicle alone. C-CASP3 and C-CASP6 levels increased after ONC+vehicle treatment but these levels remained unchanged after ONC+4000 ng/ml z-VDVAD treatment. C-CASP7 and C-CASP8 levels were much lower and did not change after ONC+vehicle, or ONC+4000 ng/ml z-VDVAD treatments. (B) and (C) Densitometry reflected these observed changes in Pro-CASP2, C-CASP2, C-CASP3, C-CASP6, C-CASP7 and C-CASP8. GAPDH was used as a loading control.

Using western blots of retinal lysates, we found that ONC+vehicle treatment not only upregulated pro-caspase-2 (Pro-CASP2) (P<0.001, ANOVA) in the retina but also significantly upregulated the levels of C-CASP2 (P<0.0001, ANOVA), particularly the p12 fragment recognised by our which our antibody, as detected by the relative band size (Figure 5A) but also after quantification by densitometry (Figure 5B). However, ONC+4000 ng/ml of z-VDVAD treatment significantly reduced C-CASP2 and the integrated density of the C-CASP2 p12 fragment (P<0.0001, ANOVA) (Figure 5A and B).

Since Pro-CASP2 can be activated by cleaved caspase-3 (C-CASP3) we probed our western blots against antibodies to C-CASP and detected the p17/19 processed fragment of C-CASP3 (Figure 5A) which was upregulated after ONC+vehicle treatment (P<0.001, ANOVA) (Figure 5A and C). However, these raised levels of C-CASP3 p17/p19 fragment were unaffected by 4000 ng/ml z-VDVAD treatment. We then probed our blots with antibodies against cleaved caspase-6 (C-CASP6), -7 (C-CASP7) and -8 (C-CASP8) to investigate if z-VDVAD non-specifically attenuated other caspases implicated in RGC survival after ONC and failed to observe any significant changes after treatments in C-CASP3, C-CASP6, C-CASP7, or C-CASP8 (Figure 5A–C).

### Inhibition of caspase-2 does not promote RGC axon regeneration after ONC

**Figure 6:**
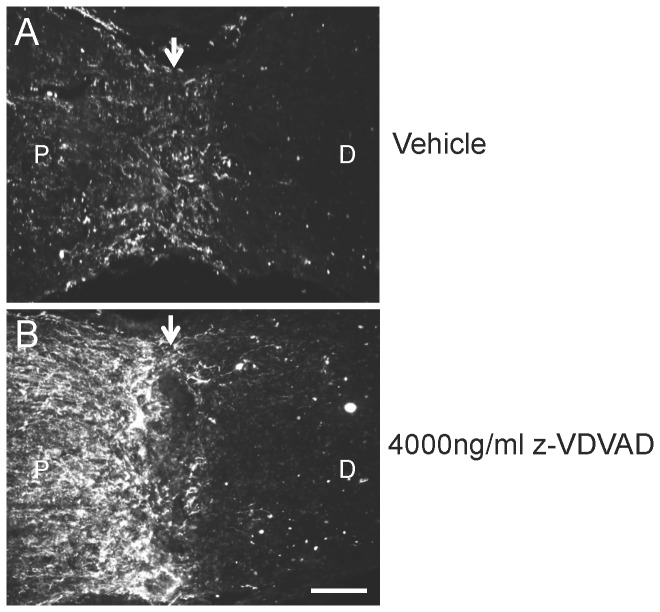
Caspase-2 inhibition did not promote RGC axon regeneration. (A) GAP-43 immunostaining in longitudinal sections of ON from eyes at 15 d after ONC, treated with ONC+vehicle showed few GAP-43^+^ axons in the proximal ON stump (P) and none leaving the lesion site (arrow) and invading the distal (D) ON segment. (B) Numerous GAP-43^+^ axons were present in the proximal segment of the transected ON after treatment with 4000 ng/ml z-VDVAD but once again no axons left the crush site (arrow) and invaded into the distal ON segment. The retina (not shown) is towards the left hand side of the image. Scale bars in (A) and (B) = 100 µm.

To investigate if suppression of caspase-2 by z-VDVAD enhanced RGC axon regeneration, we stained longitudinal sections of ON with antibodies against GAP-43 to detect regenerating axons. In ONC+vehicle treated rats, there were few surviving axons in the proximal ON segment between lesion site and eye at 15 d while no axons extended across the lesion into the distal ON segment (Figure 6A). However, in sections of ON treated with ONC+4000 ng/ml z-VDVAD greater numbers of GAP-43^+^ RGC axons were seen in the proximal ON segment, reflecting RGC survival, but no axons traversed the crush site to enter the distal ON segment (Figure 6B). Taken together, these data showed that caspase-2 inhibition promotes significant RGC survival but not axon regeneration.

## Discussion

The data presented in this study confirm our previous findings that caspase-2 is an important regulator of RGC apoptosis induced after ONC [7]. We report that caspase-2 is specifically activated in RGC and that significant RGC survival is promoted after pharmacological inhibition of caspase-2 with z-VDVAD, but no RGC axon regeneration occurs. These results complement those of our previous study with a gene silencing approach [7], demonstrating that caspase-2 is a key orchestrator of RGC apoptosis.

### Caspase-mediated RGC apoptosis

Caspase-3, -6, -8 and -9 activities all play a role in RGC apoptosis after ON injury [1], [15], [16]. Using inhibitors besides caspase-2, RGC neuroprotection ranged from 30–60% at 15 d after ONC [16], [43], [44], with caspase-8 inhibitors supporting the survival of nearly 60% RGC compared to intact controls [16]. Inhibition of caspase-2 using a chemically stabilised siRNA at 7 d after ONC rescued 98% RGC from apoptosis [7]. In the current study, pharmacological inhibition of caspase-2 with repeated delivery of z-VDVAD over a 15 d period after ONC protected 60% RGC from apoptosis, corroborating our earlier study that caspase-2 is an important regulator of RGC apoptosis [7]. We have not established the long-term efficacy of z-VDVAD on RGC neuroprotection, however, the advantage of z-VDVAD is that it is an already available, small molecule inhibitor that may be used immediately in clinical trials. Our results are similar to those of Monnier et al. (2011) [16] who reported similar levels of RGC rescue at 14 d after ONC and ON transection with a similar intravitreal delivery regime, using a caspase-8 and caspase-6 inhibitor, respectively.

Although caspase-3 inhibition rescued ∼35% RGC from death [15], we localised C-CASP3 not in RGC but in neurons of the INL [7], suggesting that caspase-3 was not a direct regulator of RGC apoptosis. In the current study, the observed elevated levels of C-CASP3 after ONC were not attenuated by z-VDVAD, suggesting that caspase-3 was not activated as a downstream effector molecule by caspase-2. Moreover, C-CASP6, C-CASP7 and C-CASP8 levels were unchanged after ONC and were also unaffected by z-VDVAD caspase-2 inhibitor suggesting that, although caspase-3 and -7 can be inhibited by the VDVAD substrates [45], C-CASP3 and C-CASP7 were not affected in this instance. We therefore, attribute the RGC protective effect of z-VDVAD to specific inhibition of caspase-2. To our knowledge this is the first study that demonstrates significant RGC protection using the pharmacological z-VDVAD inhibitor and suggests that despite these inhibitors showing some promiscuity, z-VDVAD in this case specifically inhibited caspase-2.

Although caspase-2 is structurally similar to an initiator caspase, its predicted cleavage specificity resembles that of the effector caspase-3 [46]. Caspase-2 shows a multifaceted participation in apoptosis after a variety of cellular stresses that include DNA damage, stimulation of death receptors, heat shock, cytoskeletal disruption and oxidative stress [27], [32]. In addition, caspase-2 deficient neurons are resistant to apoptosis induced by β-amyloid [31], [32], while activation of caspase-2 occurs in hippocampal neurons after transient global ischemia [47]. Moreover, caspase-2 is expressed in the RGC of ischemic retinae and the reported neuroprotective effect of intravitreal BDNF administration was associated with diminished caspase-2 immunoreactivity [34], [35]. Given this body of evidence for the involvement of caspase-2 in RGC apoptosis, the results of our previous [7] and current studies suggest that RGC death is orchestrated directly by caspase-2 after ON injury, a proposition that is supported by the observed absence of caspase-3 in RGC [7]. Similarly, few RGC were immunopositive for C-CASP6 and C-CASP8 (VV and ZA, unpublished observations), while no significant changes in the levels of C-CASP6 and C-CASP8 were observed after z-VDVAD treatment. Furthermore, significant RGC protection was observed in our current study after caspase-2 inhibition with z-VDVAD treatment without significant reductions in other potential effector caspases such as caspase-3 and -7.

### Caspase-2 inhibition does not promote RGC axon regeneration

Although there are early reports that caspase inhibition does not prevent axon degeneration [48], [49], recent evidence shows otherwise. For example, caspase-6 has been implicated as a downstream regulator of axon degeneration initiated by p75^NTR^ and β-amyloid precursor protein and death receptor 6 [50], [51], while inhibition of caspase-6 and -8 promotes RGC axon regeneration after ON injury [16]. This latter observation has been explained by demonstrations that caspase-6 predominantly degrades nuclear and cytoskeletal components and triggers microtubule destabilization [24], [52], [53] which may contribute to the failure of RGC axon regeneration [53]. Similarly, caspase-8 acts on molecules that regulate cytoskeletal dynamics, including p21-activated kinase (PAK) [54] and Rho-associated protein kinase (ROCK) [55], and may therefore contribute to the failure of RGC axon regeneration [53]. However, we did not observe RGC axon regeneration after intravitreal delivery of z-VDVAD caspase-2 inhibitor, suggesting that caspase-2 does not interact with components of the signalling cascades in which caspase-6 and -8 regulates cytoskeletal dynamics.

In conclusion, our results demonstrate that caspase-2: (1), plays a critical role in RGC apoptosis and that inhibition of caspase-2 significantly rescues RGC from death; (2), triggers RGC apoptosis in a manner that is distinct to that elicited by caspase-6 or -8; and (3), protects RGC from apoptosis after visual trauma and injury, including glaucoma [56] and optic neuropathies [57]. These results imply that targeting caspase-2 activation may be a useful therapeutic in the fight against diseases in which RGC are specifically lost, e.g. glaucoma, optic nerve injury and optic neuritis.
